# Is Hyperthyroidism a Possible Etiology of Early Onset Dementia?

**DOI:** 10.7759/cureus.10603

**Published:** 2020-09-22

**Authors:** Christopher Joy Mathew, Merin Tresa Jose, Abeer O Elshaikh, Lisa Shah, Robert Lee, Ivan Cancarevic

**Affiliations:** 1 Medicine, California Institute of Behavioral Neurosciences & Psychology, Fairfield, USA; 2 Family Medicine, California Institute of Behavioral Neurosciences & Psychology, Fairfield, USA; 3 Internal Medicine, California Institute of Behavioral Neurosciences & Psychology, Fairfield, USA; 4 Family and Community Medicine, Smt. Nathiba Hargovandas Lakhmichand Municipal Medical College, Ahmedabad, IND; 5 Surgery, California Institute of Behavioral Neurosciences & Psychology, Fairfield, USA

**Keywords:** hyperthyroidism, dementia, thyroid disorders, hyperthyroidism and dementia, mental health and endocrine diseases, hyperthyroidism and mental health, early-onset dementia, alzheimer's disease, treatment of hyperthyroidism, thyroid dysfunction

## Abstract

Dementia, a disabling syndrome of the elderly characterized by the decline in memory and cognition, is increasing in incidence and affects not only the individual but also their family and close ones. Hyperthyroidism can mimic many other diseases and untreated hyperthyroidism can lead to adverse problems of various systems including the heart, bones, muscles, menstrual cycle, and fertility. In this article, we have tried to evaluate the association between hyperthyroidism and dementia, as well as the impact of hyperthyroidism management in the treatment and prevention of dementia. Studies available in the PubMed database have been used, excluding animal studies and including studies of adults above the age of 50. The analysis of studies reveals that thyroid dysfunction can lead to cognitive impairment. It has not been able to prove that hyperthyroidism can lead to an earlier onset of dementia. But subclinical hyperthyroidism, thyroid-stimulating hormone (TSH) levels below the normal range, and high free thyroxine (T4) levels increase the risk of dementia among the elderly. The possible mechanisms involved in this association have also been discussed. Thus, we concluded that it is essential to detect and manage hyperthyroidism at an earlier stage since hyperthyroidism increases the risk of dementia. The possibility of using antithyroid treatment in euthyroid dementia is yet to be studied extensively.

## Introduction and background

Graves' disease is the most common cause of hyperthyroidism, followed by toxic nodular goiter. Other important causes of thyrotoxicosis include thyroiditis, iodine-induced, and drug-induced thyroid dysfunction, and factitious ingestion of excess thyroid hormones [1]. Symptoms mimicking many other diseases often result in inaccurate or untimely diagnoses and management. The American Association of Clinical Endocrinologists (AACE) estimated that in the United States approximately 13 million people, or 4.78% of the population, have undiagnosed thyroid dysfunction [2]. Adverse problems with the heart, bones, muscles, menstrual cycle and fertility can occur if hyperthyroidism is left untreated. Enhancing positive coping strategies and social support is important to improve mental health in Graves’ disease patients [3]. The quality of life of thyroid patients is substantially impaired over a wide range of aspects.

Dementia can be described as a syndrome resulting in the progressive decline of memory, cognition along with changes in the behavioral pattern. The disease evolution and progression not only affect the individual but also affects their family and close ones. By mid-century, the number of people living with Alzheimer’s disease in the United States is projected to grow to 13.8 million, fuelled in large part by the aging Baby Boomer generation [4]. Today, someone in the country develops Alzheimer’s disease every 66 seconds [4]. By 2050, every 33 seconds, one new case of alzheimer's is predicted to develop, leading to nearly one million new cases per annum [4]. Worldwide, around 50 million people have dementia, and there are nearly 10 million new cases every year [5]. The prevalence of dementia is expected to rise with the aging of our population for decades to come [6]. This can be overwhelming and significantly affect the quality of life causing marked distress in both the individuals, family, and also society.

The essential rights and freedoms available to others are frequently denied to people with dementia. In many countries, physical and chemical restraints are used extensively in care homes for older people and acute-care settings [5]. According to World Health Organization (WHO), young-onset dementia (defined as the onset of symptoms before the age of 65 years) accounts for up to 9% of cases [5]. Three main consequences of dementia are functional impairment and, in some cases, also mood disorders and psychosis [7]. Physical, emotional, and financial pressures can cause great stress to families and carers, and support is required from the health, social, financial, and legal systems. Early interventions are required for reducing modifiable risk factors for dementia as well as controlling medical conditions linked to dementia. Due to altered hormone levels, patients with endocrine diseases often present with other systemic and neuropsychiatric symptoms. Irreversible brain damage can occur due to delays within the diagnosis and treatment of these patients. Therefore, it's imperative for clinicians to carefully rule out the likelihood of latent endocrine diseases when treating patients with dementia. Hyperthyroidism must be considered whenever there is a change in an older person's physical or mental functioning [8].

Despite more than two decades of research on the prevention and treatment of dementia and aging-related cognitive decline, the causal association of various risk factors and highly effective preventive and therapeutic strategies remain elusive. The impact of early detection and thereby the management of hyperthyroidism in tackling dementia and cognitive decline is still unclear and yet to be studied. In this article, we are going to explore whether the current literature provides evidence for the association between hyperthyroidism and dementia as well as the impact of hyperthyroidism management in the treatment and prevention of dementia. Articles were selected from all the currently available literature in the PubMed database, based on its relevancy and detail. Studies of people above the age of 50 have been included and animal studies have been excluded.

## Review

Association of thyroid disorders and dementia/cognition

Thyroid hormones are essential for adequate cognitive functioning and behavior. Both subclinical and clinical thyroid disorders have been studied to bring out the causal association of thyroid in dementia. Alzheimer's disease (AD), frontal temporal lobe dementia, and Lewy body dementia caused by primary degeneration of the brain comprise about 70% of cases of primary dementia [9]. Effects of low dose L-triiodothyronine administration on mental, behavioral, and thyroid status in elderly subjects were analyzed by T Mori et al. in forty-four subjects [10]. The Dementia Rating (DR) score measured before and immediately after the study revealed that triiodothyronine (T3) administration to the elderly subjects was associated with behavioral improvement in some individuals, but the intellectual ability as assessed by the DR score in those with low T3 or elevated reverse triiodothyronine (rT3) were hardly improved by passive T3 elevation [10]. The effect of normalization of an isolated increase in thyroid stimulating hormone (TSH) on the neuropsychological profile of patients showed that it results in statistically significant improvement of cognitive function -- verbal, visual, and general memory [11]. In 2004, Robert A Stern et al. published a preliminary study of the relationship between thyroid status and cognitive and neuropsychiatric functioning in euthyroid patients with alzheimer's dementia. The results showed that lower concentrations of free thyroxine (FT4) are related to fear and fatigue in euthyroid individuals with AD. But thyroid hormones did not show a significant relationship with cognition [12]. A prospective longitudinal study by Bu B Yeap et al. concluded that higher FT4 levels predict new-onset dementia (as defined by International Classification of Disease [ICD] codes) in older men, independently of conventional risk factors for cognitive decline. 4.3% of the participants were diagnosed for the first time with dementia and there was no association between TSH quartiles and incident dementia. 11% increased risk of new-onset dementia per 1 pmol/liter increase in FT4, P = 0.005; quartiles Q2-4 vs. Q1: adjusted hazard ratio = 1.76, 95% confidence interval = 1.03-3.00, P = 0.04 [13]. Dhiãnah Santini de Oliveira Chachamovitz et al. investigated the association between cognitive impairment or depressive symptoms and low-normal serum TSH in elderly subjects. Low-normal TSH was not associated with a higher prevalence of dementia, but in elderly subjects without dementia, low TSH was associated with worsening cognition [14]. Carole Rieben et al. analyzed prospective cohort studies and concluded that in random-effects models, the pooled adjusted risk ratio for dementia is higher in subclinical hyperthyroidism -1.67 (95% confidence interval, 1.04; 2.69) than in subclinical hypothyroidism vs. euthyroidism -1.14 (95% confidence interval, 0.84; 1.55), both without evidence of significant heterogeneity (I2 = 0.0%). Subclinical thyroid disorders are not associated with a faster decline in mini-mental state examination over time [15].

Low dose L-triiodothyronine administration in the elderly led to behavioral improvement but not intellectual ability. The neuropsychological profile showed statistically significant improvement of cognitive function -- verbal, visual, and general memory with normalization of isolated increase in TSH. It suggests that normal thyroid function is important for improved cognition and behavior in elderly people. Euthyroid patients with Alzheimer's disease studied, did not show any significant relation between thyroid hormones and cognition but lower free thyroxine levels were associated with neuropsychiatric symptoms such as fear and fatigue. Even though some studies ruled out an association between cognition and thyroid hormones, a more statistically significant conclusion is that cognition is impaired with thyroid dysfunction. High levels of FT4 were found to predict the incidence of dementia in elderly people but TSH quartiles were not associated with the prediction of dementia. Another study also revealed that low normal serum TSH was associated with worsening cognition but no association with a higher prevalence of dementia. Both studies revealed that low serum TSH is not useful in predicting dementia and its prevalence. However, recent studies give evidence that subclinical hyperthyroidism increases the risk for dementia rather than subclinical hypothyroidism. A substantial body of evidence is present to support the association between thyroid dysfunction and cognition, but larger and more detailed studies are required to validate the association between thyroid dysfunction and dementia. Hypothyroidism and subclinical hyperthyroidism, both have been related to cognitive impairment and dementia.

Hyperthyroidism and increased risk of dementia

An association between elevated thyroid hormones and increased risk of dementia has long been debated. The effects of thyroid hormones under physiological and pathological conditions have been extensively studied during the last two centuries and significant morbidity and mortality are associated with overt thyroid dysfunction [16]. Kristen M Geroge et al. studied 12,481 participants and found a higher risk of dementia (hazard ratio [HR] [95% CI]: 1.40 [1.02 - 1.92]) among the overt hyperthyroid patients than the euthyroid participants. The dementia risk is increased by abnormal thyroid hormone levels (high or low) and autoimmunity from autoimmune thyroid disease (AITD) [17]. In a study of the psychiatric population on admission, H.A. Oomen et al. noticed more prevalence of affective disorders in thyroperoxidase antibody (TPO-Ab) positive patients and patients with a low serum TSH, but no differences in the prevalence of dementia, schizophrenia, or other psychiatric illnesses [18]. In a random sample of 1,843 participants, aged 55 years and over, from the population-based prospective Rotterdam Study, Kalmijn S et al. found that more than threefold increased risk of dementia (relative ratio **[**RR**]** = 3.5, 95% CI: 1.2-10.0) and Alzheimer's disease (RR = 3.5, 95% CI: 1.1-11.5) after adjustment for age and sex, is seen in persons with decreased TSH levels at baseline. A positive relation is found between T4 levels and the risk of dementia (RR per standard deviation (SD) increase = 2.9, 95% CI: 0.7-12.2) among persons with reduced TSH levels [19]. Döbert N et al. analyzed different types of dementia and their correlation with thyrotropin (TSH) and thyroid antibodies. An increased probability of having dementia, especially vascular dementia was found among those with decreased or borderline TSH values [20]. Isabela M Benseñor et al. suggested a consistent association in the São Paulo Ageing & Health Study (SPAH) between dementia and subclinical hyperthyroidism [21]. Earn H Gan et al. in a systemic review, found evidence supporting the association between cognitive impairment and subclinical hyperthyroidism, but the mechanistic explanation is not available [22]. Bu B Yeap et al. did a prospective longitudinal study that excluded men with overt thyroid disease or dementia or standardized mini-mental state examination scores below 24. The assay of circulating TSH and free T4 (FT4) concluded that independent of conventional risk factors for cognitive decline, new-onset dementia in older men is predicted by higher FT4 levels [13]. Moon JH et al. in a population-based prospective study of 313 euthyroid non-demented elderly participants, tried to find the relation between the risk of dementia and thyroid function. After a five-year follow-up evaluation (mean age 72.5 ± 6.9 years), the risk of cognitive impairment including mild cognitive impairment (MCI) and dementia in elderly subjects was independently associated with lower serum TSH levels within the reference range [23]. A cross-sectional hospital-based study was conducted by Agarwal R et al. on AD/vascular dementia (VaD) diagnosed subjects (114 AD patients with mean age: 65 years, 35 VaD patients with mean age: 62 years). A routine screening test, free T3, free T4, and TSH were assessed in the cases and 105 control subjects with mean age - 62 years, which revealed subclinical hyperthyroidism and AD to have a consistent association [24]. A meta-analysis of 11 studies by Yue Wu et al. with a total of 24,952 participants, established relationships between dementia and the per SD increment of free thyroxine (FT4) (RR = 1.08, 95% CI 1.00-1.17) and thyroid-stimulating hormone (TSH) (fixed RR = 0.91, 95% CI 0.84-0.99) [25]. An increased risk of dementia (fixed RR = 1.60, 95% CI 1.27-2.00) was found with TSH levels below the normal range and with higher FT4 levels. But low or high categories of TSH in men did not show any association with the risk of dementia [25].

Studies with a larger sample size have shown statistically significant evidence of hyperthyroidism increasing the risk of dementia. Among the various thyroid dysfunctions, both hypo and hyperthyroidism, and autoimmune thyroid diseases are associated with cognitive impairment. Hyperthyroid conditions are associated with a greater risk of dementia compared to euthyroidism. Rotterdam's study has shown a threefold increase in dementia and Alzheimer’s disease in those with reduced TSH levels. The majority of the studies have been consistent with the finding of subclinical hyperthyroidism increasing the risk of dementia. Only one study has reported that low TSH values and positive TPO-Ab are not associated with a higher prevalence of dementia. A higher free T4 level was found to be predictive of new-onset dementia in the elderly. Thus, TSH levels below the normal range and high free T4 levels associated with increased risk of dementia can demand an increased need for early detection and management of both clinical and subclinical hyperthyroidism. However, substantial evidence is not available to conclude that hyperthyroidism can lead to the early onset of dementia. Further analysis is required to evaluate an earlier onset of dementia in hyperthyroid individuals compared to euthyroidism.

Possible mechanisms causing dementia in thyroid dysfunction

Much insight is not available into the possible mechanisms of increased risk in patients with thyroid dysfunction. As hypothyroidism and hyperthyroidism are being investigated to establish a relation with dementia, it is important to understand the underlying pathology responsible for the correlation and thus modify the management of dementia. Belandia B et al. published that thyroid hormone regulates the gene expression of the amyloid-beta protein precursor (AβPP) [26,27]. And O’Barr SA et al. published that in both in vitro and in vivo models, thyroid hormone regulates endogenous amyloid-beta precursor protein gene expression and processing [28]. Evidence of thyroid dysfunction leading to dementia by an underlying vascular mechanism can be obtained, as vascular risk factors and cardiovascular disease increasing the risk of Alzheimer’s disease incidence have been analyzed [29, 30]. Also, cardiovascular risk is increased by both clinical and sub-clinical thyroid dysfunction [31-33]. De Jong FJ et al. in the Rotterdam Study found an association between polymorphisms in type 1 and 2 deiodinase genes and iodothyronine levels in the elderly but no association of these polymorphisms with early neuroimaging markers for AD [34]. Bianchi G et al. confirmed the presence of oxidative stress and decreased antioxidant metabolites in hyperthyroid patients which may be responsible for degenerative changes in the brain [35]. Studies found that thyroxine can generate oxidative stress and damage neurons [36]. Zaldy S Tan et al. analyzed the enhanced risk of Alzheimer’s disease due to the effects of thyroid hormone on cerebral amyloid processing, neurodegeneration. Vascular and thyrotropin-mediated mechanisms were also evaluated [37]. Bobban Subhadra et al. suggested that in Alzheimer’s disease, the up-regulation of neuroserpin which inhibits tissue plasminogen activator (tPA) activity and affects the degradation and clearance of amyloid-beta and its plaques from the brain may result from activation of the thyroid hormone response system [38]. By using neurite outgrowth studies Oldham Ce et al. demonstrated that T3 treatment enhanced neurite outgrowth in NeuroScreen 1 Cells (NS-1 cells) in a time- and dose-dependent manner. Also, the T3 treatment impacted the splicing of tau exon 10 in the direction of producing more tau molecules that excluded the exon (tau 3R) [39]. Further, Lun-Xi Li found that hyperthyroidism participants had significantly higher total tau protein levels than in their euthyroid counterparts. The serum concentrations of free T3 (FT3) and FT4 are positively associated with the level of circulating total tau [40].

Several studies explored possible biological mechanisms to explain the association between thyroid function and dementia. Certain studies stated that thyroid hormones are not involved in developing AD, but the association between iodothyronine levels and brain atrophy may be reflecting comorbidity. AD is early affected by the cholinergic system, and evidence points to the cholinergic system being influenced by thyroid hormones. Development of AD was also found to be contributed by low central nervous system thyroid hormone levels by directly increasing amyloid-β protein precursor (AβPP) expression and consequently, amyloid-β peptide and amyloid-β levels. Enhanced neuronal death by exposure to thyroid hormone and hyperthyroidism causing oxidative stress and decreased anti-oxidant metabolites has also been shown. Thyroid function may indirectly affect AD through the increased vascular risk factors. Another possible mechanism can be a serum concentration of tau which may be involved in the pathogenesis of AD. The possible mechanisms are presented in Table 1. Further studies are required to confirm the role of T3 levels in AD development as it can suggest a potential therapeutic target of AD via hyperthyroidism therapy.

**Table 1:** Possible mechanisms causing dementia in thyroid dysfunction AβPP: amyloid-beta protein precursor

Possible mechanisms causing dementia in thyroid dysfunction
Low thyroid hormones increasing AβPP expression and consequently, amyloid-β peptide and amyloid-β levels
Reduced degradation and clearance of amyloid-beta plaques from brain, due to the up-regulation of neuroserpin by activation of thyroid hormone response
Oxidative stress and decreased antioxidant metabolites in hyperthyroid patients causing degenerative changes in the brain and neuronal death
Thyroid dysfunction increasing vascular risk factors which lead to alzheimer’s disease
Higher total tau protein levels seen in hyperthyroid patients

Treatment of hyperthyroidism in dementia prevention

The efficient management of hyperthyroidism including subclinical hyperthyroidism has various indications. Newer indications of anti-thyroid treatment as in the role of improving cognitive function are yet to be studied. VA Portnoi reported a case of hyperthyroidism with a dementia-like presentation, which on treating hyperthyroidism, features of dementia resolved [41]. T Mori et al. analyzed the effect of passive T3 elevation in the elderly by administering low dose L-triiodothyronine and found that even though the behavioral improvement was noted, no improvement was found in the dementia rating score [10]. UG Cunha followed up patients with potentially reversible dementia and found that no significant improvement in the mental status is observed even after proper treatment of hypothyroidism, hyperthyroidism, and vitamin B12 deficiency [42]. There was a persistence of decline in cognition [42]. FI Martin found clinical improvement and normal results of thyroid function tests in 35 patients (21 had dementia) among the 47 hyperthyroid patients who were managed with antithyroid drugs and radioactive iodine [43]. T Fukui reported a case of hyperthyroid dementia which on treatment with a beta-blocker and later methimazole showed that restoration of the euthyroid state improved behavior, memory, and constructive abilities [44]. Yuichiro Ii et al. analyzed a case of hyperthyroidism which presented as transient dementia. Memory disturbance and abnormal behavior were resolved when the euthyroid state was attained [45]. Milos Zarković reported that variations in thyroid hormone concentration can cause reversible cognitive defects which improve on treating the thyroid dysfunction [46]. Gaetana Napolitano et al. reported that mitochondrial reactive oxygen species and oxidative damage seen in hyperthyroid patients can be reduced by vitamin E supplementation, and thereby preserve the cell function to avoid neurodegenerative disorders [47]. Bernadette Biondi analyzed the need for treating subclinical hyperthyroidism and reported that cardiovascular diseases, dementia, and bone loss/fracture are associated with severe subclinical hyperthyroidism. A serum TSH level above 10 mIU/L is to be treated to decrease cardiovascular risks [48].

Disease presentation in the elderly can be highly variable and there have been incidents of treatable and reversible disease conditions being misdiagnosed as dementia. Hyperthyroidism is one such condition that presents as dementia and treatment of the thyroid dysfunction in such cases will reverse the cognitive impairment. Therefore, timely thyroid function screening and treatment in the aged population can prevent the avoidable/treatable cognitive impairment and transient dementia. The results regarding the effectiveness of treating hyperthyroidism in the management and prevention of dementia are heterogeneous. Some studies showed that the management of hyperthyroidism does not revert dementia into a normal state and shows a progressive decline. While other studies revealed improvement of hyperthyroid dementia on treating the thyroid dysfunction. It was also found that the oxidative stress and damage in hyperthyroid patients can be reduced by vitamin E intake, which preserves cell function and thereby controls the progression of dementia. There is still no clear evidence to indicate the use of anti-thyroid drugs in the treatment of dementia except for thyroid dysfunction induced dementia. More detailed studies are required to analyze the use of thyroid drugs in euthyroid dementia patients. However appropriate treatment for hyperthyroidism is always essential as it can lead to serious comorbidities.

Limitations

The limited access to data from the PubMed database and the need to draw conclusions from diverse studies was a constraint in this study. The data presented was not representative of all age groups. Conflicting study results were presented in different studies. Some of the studies included had a small sample size and did not yield statistically significant results. The quality assessment could not be carried out for all the studies used. Also, there was a lack of previous research studies on thyroid dysfunction and younger onset dementia. Sufficient studies were not available to analyze the effectiveness of antithyroid treatment in dementia.

Future recommendations

Further research and studies are to be conducted involving the younger age population to detect young onset dementia, so that data representative of different age groups becomes available. More detailed studies involving larger sample size are to be performed, yielding statistically significant results. The risks involved in antithyroid treatment of euthyroid dementia patients are to be evaluated. Also, the advantages and effectiveness of thyroid medications in prevention of dementia has to be studied further.

## Conclusions

Dementia, a syndrome of progressive decline in memory, cognition, and behavioral changes, gravely reduces the quality of life in the elderly. Thyroid dysfunction, both hypothyroidism and hyperthyroidism have shown to cause severe comorbidities, including cognitive impairment, and we wanted to find out if hyperthyroidism increases the risk of dementia. Subclinical hyperthyroidism increases the risk for dementia rather than subclinical hypothyroidism, and improved cognition and behavior in elderly people require normal thyroid function. TSH levels below the normal range and high free T4 levels are associated with an increased risk of dementia and can demand the need for early detection and management of both clinical and subclinical hyperthyroidism. However, the results regarding the effectiveness of treating hyperthyroidism in the management and prevention of dementia are heterogeneous. Lack of clear evidence to confirm the incidence of dementia at a younger age in hyperthyroid patients, and the benefits of antithyroid treatment in euthyroid dementia necessitate further detailed study.

## References

[1] De Leo S, Lee SY, Braverman LE (2016). Hyperthyroidism. Lancet.

[2] Garber JR, Cobin RH, Gharib H (2012). Clinical practice guidelines for hypothyroidism in adults: cosponsored by the American Association of Clinical Endocrinologists and the American Thyroid Association. Endocr Pract.

[3] Chen DY, Schneider PF, Zhang XS, He ZM (2012). Mental health status and factors that influence the course of Graves' disease and antithyroid treatments. Exp Clin Endocrinol Diabetes.

[4] Alzheimer's Association (2016). 2016 Alzheimer's disease facts and figures. Alzheimers Dement.

[5] (2020). Dementia. https://www.who.int/news-room/fact-sheets/detail/dementia.

[6] Radue R, Walaszek A, Asthana S (2019). Chapter 24 - Neuropsychiatric symptoms in dementia. Handb Clin Neurol.

[7] Volicer L (2018). Behavioral problems and dementia. Clin Geriatr Med.

[8] Gambert SR (1995). Hyperthyroidism in the elderly. Clin Geriatr Med.

[9] Brookmeyer R, Gray S, Kawas C (1998). Projections of Alzheimer's disease in the United States and the public health impact of delaying disease onset. Am J Public Health.

[10] Mori T, Inoue D, Kosugi S (1988). Effects of low dose L-triiodothyronine administration on mental, behavioral, and thyroid states in elderly subjects. Endocrinol Jpn.

[11] Jensovský J, Spacková N, Hejduková B, Růzicka E (2000). Vliv normalizace izolovanĕ zvýseného TSH na neuropsychologický profil pacientů [Effect of normalization of an isolated increase in TSH on the neuropsychological profile of patients]. Cas Lek Cesk.

[12] Stern RA, Davis JD, Rogers BL (2004). Preliminary study of the relationship between thyroid status and cognitive and neuropsychiatric functioning in euthyroid patients with Alzheimer dementia. Cogn Behav Neurol.

[13] Yeap BB, Alfonso H, Chubb SA (2012). Higher free thyroxine levels predict increased incidence of dementia in older men: the Health in Men Study. J Clin Endocrinol Metab.

[14] Chachamovitz DS, Vigário Pdos S, Silva SO (2016). Does low-normal serum TSH level adversely impact cognition in elderly adults and might methimazole therapy improve outcomes?. Endocr J.

[15] Rieben C, Segna D, da Costa BR (2016). Subclinical thyroid dysfunction and the risk of cognitive decline: a meta-analysis of prospective cohort studies. J Clin Endocrinol Metab.

[16] Boelaert K, Franklyn JA (2005). Thyroid hormone in health and disease. J Endocrinol.

[17] George KM, Lutsey PL, Selvin E (2019). Association between thyroid dysfunction and incident dementia in the atherosclerosis risk in communities neurocognitive study. J Endocrinol Metab.

[18] Oomen HA, Schipperijn AJ, Drexhage HA (1996). The prevalence of affective disorder and in particular of a rapid cycling of bipolar disorder in patients with abnormal thyroid function tests. Clin Endocrinol (Oxf).

[19] Kalmijn S, Mehta KM, Pols HA (2000). Subclinical hyperthyroidism and the risk of dementia. The Rotterdam study. Clin Endocrinol (Oxf).

[20] Döbert N, Hamscho N, Menzel C (2003). Subclinical hyperthyroidism in dementia and correlation of the metabolic index in FDG-PET. Acta Med Austriaca.

[21] Benseñor IM, Lotufo PA, Menezes PR, Scazufca M (2010). Subclinical hyperthyroidism and dementia: the Sao Paulo Ageing & Health Study (SPAH). BMC Public Health.

[22] Gan EH, Pearce SH (2012). The thyroid in mind: cognitive function and low thyrotropin in older people. J Clin Endocrinol Metab.

[23] Moon JH, Park YJ, Kim TH (2014). Lower-but-normal serum TSH level is associated with the development or progression of cognitive impairment in elderly: Korean Longitudinal Study on Health and Aging (KLoSHA). J Clin Endocrinol Metab.

[24] Agarwal R, Kushwaha S, Chhillar N (2013). A cross-sectional study on thyroid status in North Indian elderly outpatients with dementia. Ann Indian Acad Neurol.

[25] Wu Y, Pei Y, Wang F, Xu D, Cui W (2016). Higher FT4 or TSH below the normal range are associated with increased risk of dementia: a meta-analysis of 11 studies. Sci Rep.

[26] Belandia B, Latasa MJ, Villa A, Pascual A (1998). Thyroid hormone negatively regulates the transcriptional activity of the beta-amyloid precursor protein gene. J Biol Chem.

[27] Latasa MJ, Belandia B, Pascual A (1998). Thyroid hormones regulate beta-amyloid gene splicing and protein secretion in neuroblastoma cell. Endocrinology.

[28] O’Barr SA, Oh JS, Ma C, Brent GA, Schultz JJ (2006). Thyroid hormone regulates endogenous amyloid-beta precursor protein gene expression and processing in both in vitro and in vivo models. Thyroid.

[29] Luchsinger JA, Reitz C, Honig LS, Tang MX, Shea S, Mayeux R (2005). Aggregation of vascular risk factors and risk of incident Alzheimer’s disease. Neurology.

[30] Newman AB, Fitzpatrick AL, Lopez O (2005). Dementia and Alzheimer's disease incidence in relationship to cardiovascular disease in the Cardiovascular Health Study cohort. J Am Geriatr Soc.

[31] Walsh JP, Bremner AP, Bulsara MK (2005). Subclinical thyroid dysfunction as a risk factor for cardiovascular disease. Arch Intern Med.

[32] Hak AE, Pols HAP, Visser TJ, Drexhage HA, Hofman A, Witteman JC (2000). Subclinical hypothyroidism is an independent risk factor for atherosclerosis and myocardial infarction in elderly women: the Rotterdam study. Ann Intern Med.

[33] Toft AD, Boon NA (2000). Thyroid disease and the heart. Heart.

[34] de Jong FJ, Peeters RP, den Heijer T (2007). The association of polymorphisms in the type 1 and 2 deiodinase genes with circulating thyroid hormone parameters and atrophy of the medial temporal lobe. J Clin Endocrinol Metab.

[35] Bianchi G, Solaroli E, Zaccheroni V (1999). Oxidative stress and anti-oxidant metabolites in patients with hyperthyroidism: effect of treatment. Horm Metab Res.

[36] Hogervorst E, Huppert F, Matthews FE, Brayne C (2008). Thyroid function and cognitive decline in the MRC Cognitive Function and Ageing Study. Psychoneuroendocrinology.

[37] Tan ZS, Vasan RS (2009). Thyroid function and Alzheimer's disease. J Alzheimers Dis.

[38] Subhadra B, Schaller K, Seeds NW (2013). Neuroserpin up-regulation in the Alzheimer's disease brain is associated with elevated thyroid hormone receptor-β1 and HuD expression. Neurochem Int.

[39] Oldham CE, Wooten CJ, Williams AB, Dixon S, Lopez D (2018). Thyroid hormone enhances neurite outgrowth in neuroscreen 1 cells. Int J Biomed Investig.

[40] Li LX, Yang T, Guo L (2020). Serum tau levels are increased in patients with hyperthyroidism. Neurosci Lett.

[41] Portnoi VA (1979). Thyrotoxicosis as a mimic of dementia and/or stroke-like syndrome. Postgrad Med.

[42] Cunha UG (1990). An investigation of dementia among elderly outpatients. Acta Psychiatr Scand.

[43] Martin FI, Deam DR (1996). Hyperthyroidism in elderly hospitalised patients: clinical features and treatment outcomes. Med J Aust.

[44] Fukui T, Hasegawa Y, Takenaka H (2001). Hyperthyroid dementia: clinicoradiological findings and response to treatment. J Neurol Sci.

[45] Ii Y, Ohira T, Narita Y, Kuzuhara S (2003). Transient dementia during hyperthyroidism of painless thyroiditis: a case report. Rinsho Shinkeigaku.

[46] Zarković M (2005). Autoimmune thyroid disease and brain. Srp Arh Celok Lek.

[47] Napolitano G, Fasciolo G, Di Meo S, Venditti P (2019). Vitamin E supplementation and mitochondria in experimental and functional hyperthyroidism: a mini-review. Nutrients.

[48] Biondi B (2020). "Is there any reason to treat subclinical hypo and hyperthyroidism?". Ann Endocrinol (Paris).

